# Healthcare Utilization in Myalgic Encephalomyelitis/Chronic Fatigue Syndrome (ME/CFS): Analysis of US Ambulatory Healthcare Data, 2000–2009

**DOI:** 10.3389/fped.2019.00185

**Published:** 2019-05-14

**Authors:** Jaeyong Bae, Jin-Mann S. Lin

**Affiliations:** ^1^Chronic Viral Diseases Branch, Division of High-Consequence Pathogens and Pathology, National Center for Emerging and Zoonotic Infectious Diseases, Centers for Disease Control and Prevention, Atlanta, GA, United States; ^2^Department of Health Policy and Management, Rollins School of Public Health, Emory University, Atlanta, GA, United States

**Keywords:** myalgic encephalomyelitis/chronic fatigue syndrome, National Ambulatory Medical Care Survey (NAMCS), co-morbidities, healthcare utilization, quality indicators of healthcare

## Abstract

**Background:** ME/CFS is a complex and disabling illness with substantial economic burden and functional impairment comparable to heart disease and multiple sclerosis. Many patients with ME/CFS do not receive appropriate healthcare, partially due to lack of diagnostic tests, and knowledge/attitudes/beliefs about ME/CFS. This study was to assess the utility of US ambulatory healthcare data in profiling demographics, co-morbidities, and healthcare in ME/CFS.

**Methods:** Data came from the National Ambulatory Medical Care Survey (NAMCS) and the National Hospital Ambulatory Medical Care Survey (NHAMCS) in the U.S. Weighted analysis was performed. We examined 9.06 billion adult visits from 2000 to 2009 NAMCS/NHAMCS data. ME/CFS-related visits were identified by ICD-9-CM code, 780.71, up to tertiary diagnosis.

**Results:** We estimated 2.9 million (95% CI: 1.8–3.9 million) ME/CFS-related visits during 2000–2009, with no statistical evidence (p-trend = 0.31) for a decline or increase in ME/CFS-related visits. Internists, general and family practitioners combined provided 52.12% of these visits. Patients with ME/CFS-related visits were mostly in their 40 and 50 s (47.76%), female (66.07%), white (86.95%), metropolitan/urban residents (92.05%), and insured (87.26%). About 71% of ME/CFS patients had co-morbidities, including depression (35.79%), hypertension (31.14%), diabetes (20.30%), and arthritis (14.11%). As one quality indicator, physicians spent more time on ME/CFS-related visits than non-ME/CFS visits (23.62 vs. 19.38 min, *p* = 0.065). As additional quality indicators, the top three preventive counseling services provided to patients with ME/CFS-related visits were diet/nutrition (8.33%), exercise (8.21%), and smoking cessation (7.24%). Compared to non-ME/CFS visits, fewer ME/CFS-related visits included counseling for stress management (0.75 vs. 3.14%, *p* = 0.010), weight reduction (0.88 vs. 4.02%, *p* = 0.002), injury prevention (0.04 vs. 1.64%, *p* < 0.001), and family planning/contraception (0.17 vs. 1.45%, *p* = 0.037).

**Conclusions:** Visits coded with ME/CFS did not increase from 2000 to 2009. Almost three quarters of ME/CFS-related visits were made by ME/CFS patients with other co-morbid conditions, further adding to complexity in ME/CFS healthcare. While physicians spent more time with ME/CFS patients, a lower proportion of ME/CFS patients received preventive counseling for weight reduction, stress management, and injury prevention than other patients despite the complexity of ME/CFS. NAMCS/NHAMCS data are useful in evaluating co-morbidities, healthcare utilization, and quality indicators for healthcare in ME/CFS.

## Background

Myalgic encephalomyelitis/chronic fatigue syndrome (ME/CFS) is a multi-system illness characterized by reduced functioning associated with fatigue that is not due to ongoing exertion and not significantly improved by rest. Minimal mental or physical exertion may trigger relapse (termed post-exertional malaise). Additional core or common symptoms include unrefreshing sleep, cognitive problems, increased symptoms when standing, and pain, but patients may experience numerous other symptoms (1–4). Several different etiologies have been investigated for ME/CFS but so far the etiology cannot be fully explained (4, 5). Experts have noted that the terminology “chronic fatigue syndrome” can trivialize this illness and stigmatize persons who experience its symptoms (6). A variety of other names have been used, including myalgic encephalomyelitis (ME), ME/CFS, chronic fatigue immune dysfunction, and most recently, systemic exertion intolerance disease (4). In 2010, the Chronic Fatigue Syndrome Advisory Committee recommended use of ME/CFS across federal agencies within the Department of Health and Human Services. The term ME/CFS will be used in this paper.

Previous prevalence estimates of ME/CFS have varied from 0.007 to 8.34% (7–16). In the community-based studies (with or without clinical assessment), the prevalence of ME/CFS was estimated to be 0.007–8.34%, and in the clinical-based studies in primary care setting the prevalence was estimated to be 0.006–3.00%. The large variation in the ME/CFS prevalence estimates may be due to differences in study methodology, such as study population composition, heterogeneity of source populations, data collection procedures, limitations in case ascertainment, different case definitions, and operational application of case definition criteria. Moreover, most prior studies took place at a small number of hospitals or clinics, or used a population sample from one state (9, 11, 13, 17, 18).

The economic burden and functional impairment associated with ME/CFS is substantial and comparable to heart disease and multiple sclerosis. ME/CFS accounts for $18–51 billion of economic costs including $9–14 billion in medical costs and $9–37 billion in lost productivity annually (19–21). Patients with ME/CFS suffer from worse functional impairment compared to cancer, diabetes, heart disease, lung disease, multiple sclerosis, and rheumatoid arthritis (15, 22). Additionally, many patients with ME/CFS are found to have additional overlapping pain conditions such as fibromyalgia, multiple chemical sensitivity, and irritable bowel syndrome (23–27). Adjusting to a chronic, debilitating illness sometimes leads to other problems, including depression, stress, and anxiety. Like patients with other chronic illnesses, many patients with ME/CFS develop depression during their illness course (28, 29). ME/CFS patients with other co-morbid conditions have poor health and worse functioning status than those without co-morbid conditions. Co-morbid or co-existing conditions may also increase the frequency of healthcare utilization including office visits and laboratory tests (27, 30) and further complicate the management of ME/CFS symptoms. Many ME/CFS patients do not receive appropriate healthcare, partially due to constraints US healthcare systems face in addressing chronic illnesses but also due to healthcare providers lack of knowledge and misaligned attitudes, and beliefs concerning ME/CFS (31, 32).

In this study, we sought to use US national healthcare data from ambulatory visits to evaluate trends from 2000 to 2009 in ME/CFS. Additionally, we characterized demographics, co-morbidities, and healthcare services/quality indicators related to ME/CFS visits.

## Methods

### Data Sources

This analysis was based on the National Ambulatory Medical Care Survey (NAMCS) (33) and the National Hospital Ambulatory Medical Care Survey (NHAMCS) (34) from 2000 to 2009. Since 1992, both surveys have been administrated by the National Center for Health Statistics (NCHS), Centers for Disease Control and Prevention (CDC) (35–38). The NCHS Research Ethics Review Board approved the protocols for both the NAMCS and NHAMCS surveys, including a waiver of the requirement for informed consent of participating patients.

In brief, NAMCS collects healthcare data provided by non-federal office-based physicians whereas NHAMCS collects healthcare data provided by non-federal hospital outpatient departments (OPDs) and hospital emergency departments (EDs). Both surveys use multistage probability sampling procedures to allow for generating nationally representative estimates of ambulatory medical care services in the United States. The patient visit was the unit of this analysis. Between 2000 and 2009, response rates were 58.9–70.4% among physicians invited to participate in NAMCS, 68.3–91.0% among hospital OPDs, and 79.5–97.0% among EDs invited to participate in NHAMCS.

This analysis included the aggregated number of 748,464 adult visits (made by patients aged 18 years or older, 9.06 billion weighted visits) from the NAMCS and NHAMCS data during 2000–2009. This included 231,984 physician patient visits (7.49 billion weighted visits), 250,821 OPD visits (691 million weighted visits), and 265,659 ED visits (879 million weighted visits).

### Measures

The US national ambulatory data includes up to three listed diagnoses (primary, secondary, and tertiary) for each visit. We classified all visits into two types: ME/CFS-related visits and non-ME/CFS-related visits. ME/CFS-related visits were identified using the International Classification of Diseases, 9th revision, Clinical Modification (ICD-9-CM) code, 780.71, up to tertiary diagnosis received in provider's diagnoses during visits. The primary outcomes of interest were: (1) co-morbidities, (2) healthcare services. We also examined the associations between types of visits and patient demographics.

#### Co-morbidities

In addition to the provider's diagnoses for patient visits, providers (for NAMCS and OPD only for NHAMCS) indicate the presence of 14 conditions even if the condition had been reported in the diagnosis box. The 14 conditions were arthritis, asthma, cancer, cerebrovascular disease, congestive heart failure, chronic renal failure, chronic obstructive pulmonary disease, depression, diabetes, hyperlipidemia, hypertension, ischemic heart disease, obesity, and osteoporosis. We considered these to be co-morbid conditions, and compared the percentages of ME/CFS and non-ME/CFS-related visits with these conditions.

#### Healthcare Services

Both NAMCS and NHAMCS surveys collected information on any service ordered or provided for patients during their visits. Our analysis included three categories of healthcare services: (1) preventive counseling/management services—asthma, diet/nutrition, exercise, family planning/contraception, growth/development, injury prevention, stress management, tobacco use/exposure, weight reduction, and other counseling, (2) diagnostic/screening services—complete blood count (CBC), glucose, glycohemoglobin (HgbA1c), lipid/cholesterol, and other blood tests, and (3) non-medication treatments—Complementary Alternative Medicine (CAM), physical therapy, psychotherapy, and other mental health counseling. We also examined the length of the patient's visit. These healthcare services have been constructed as quality indicators in other illnesses along with other illness-specific quality indicators based on the Institute of Medicine's broad criteria of clinical importance, scientific soundness, and feasibility for indicator selection (39, 40).

Other variables used in this study were: (1) patient demographics- age, sex, race/ethnicity, source of payment source including insurance type, and tobacco use (current or not), (2) vital signs—body mass index (BMI), and blood pressure (BP), and (3) physician/clinic information—geographic region (Northeast/Midwest/South/West), metropolitan statistical area (MSA or not), physician specialty (NAMCS only), clinic type (OPD only), physician practice characteristics (NAMCS only; solo practitioner physician practice characteristics employment status, ownership, office type), use of electronic medical records (EMRs), and referral status.

### Statistical Analysis

Statistical analyses were performed using STATA version 12 (Stata Corporation, College Station, TX). To report national estimate, the “svy” command designed for multistage weighted probability surveys such as NAMCS and NHAMCS were utilized. The NCHS analytical guidelines establish the legitimacy of combining multiple years of data from the NAMCS and NHAMCS surveys. Comparisons of NAMCS and NHAMCS suggested limited differences in the percentage estimates of ME/CFS-related visits annually. We, therefore, combined the two surveys for subsequent analyses between ME/CFS-related and non-ME/CFS-related visits.

The Pearson correlation test was used to analyze the percentage trend of ME/CFS-related visits during 2000–2009. In addition to national estimate of ME/CFS-related visits, we examined bivariate associations of ME/CFS-related visits with the following health and healthcare outcomes: (1) co-morbidities, (2) quality of healthcare such as health education services and length of patient's visit, (3) other variables such as patient and physician/clinic information, and vital signs.

## Results

### Demographic Characteristics of ME/CFS-Related Visits

Table 1 shows the demographics for overall visits to physician offices, hospital outpatient departments, and emergency departments. Among 784,464 visits made by patients ≥18 years of age in the 2000–2009 NAMCS and NHAMCS datasets, we identified 130 visits (unweighted counts) indicating ICD-9-CM code, 780.71 for ME/CFS. After appropriate weighting, the estimated number of visits made by patients with ME/CFS in the United States over the 10 years period was 2.9 million [95% Confidence interval (CI): 1.8–3.9 million]. ME/CFS patient visits were mostly in the fourth and fifth decade age group (48.76%), female (66.07%), white (86.95%), metropolitan/urban residents (92.04%), and insured (87.26%). There were no statistically significant difference on the distribution of age, sex, race, residential area, and insurance between ME/CFS and non-ME/CFS related visits. Of the ME/CFS-related visits to office-based physicians, 52.12% were to general/family practitioners and internists. Obstetrics and gynecology, psychiatry, and neurology combined only accounted for <10% while all other specialties accounted for 38.13% ME/CFS patient visits. Among ME/CFS-related visits to hospital outpatient departments, general medicine clinics accounted for most of the visits (72.38%). Compared to non-ME/CFS visits, a slightly higher rate of adopting electronic health record (EHR) systems was observed in ME/CFS-related visits (35.91 vs. 42.95%).

**Table 1:** Patient and practice demographic characteristics of ambulatory adult visits in USA, 2000–2009.

**Variables**	**All visits**	**ME/CFS visits**	**Non-ME/CFS visits**	***P*-value^**a**^**
Unweighted no. of visits	748,464	130	748,334	
Weighted no. of visits	9,061,664,246	2,911,161	9,058,753,085	
Practice setting (%)				
Office based (NAMCS)	7,491,099,961 (82.67%)	2,723,988 (93.57%)	7,488,375,973 (82.66%)	
Outpatient department (NHAMCS-OPD)	690,679,940 (7.62%)	119,850 (4.12%)	690,560,090 (7.62%)	
Emergency department (NHAMCS-ED)	879,884,345 (9.71%)	67,323 (2.31%)	879,817,022 (9.71%)	
Physician Specialty^1^ (%)				0.382
General and family practice	24.97%	35.86%	24.97%	
Internal medicine	18.82%	16.26%	18.82%	
Obstetrics and gynecology	9.33%	2.06%	9.33%	
Psychiatry	3.15%	4.72%	3.15%	
Neurology	1.56%	2.96%	1.56%	
All other	42.18%	38.13%	42.18%	
Clinical Type^2^ (%)				0.001
General medicine	65.75%	72.38%	65.75%	
Surgery	13.48%	16.03%	13.48%	
Pediatric	1.06%	0.00%	1.06%	
Obstetrics and Gynecology	10.45%	0.13%	10.45%	
Substance abuse	0.92%	0.00%	0.92%	
Other	8.34%	11.46%	8.34%	
	(n: 6,075,856,067)	(n: 1,673,624)	(n: 6,074,182,443)	
EHR use^3^ (%)	35.91%	42.95%	35.91%	0.462
Age, mean (SD)	52.65 (18.89)	48.56 (11.60)	52.66 (18.89)	0.077
Age, median (range)	47 (18–100)	47.5(20–89)	47 (18–100)	
Age Group (%)				0.440
18 to 29	13.81%	15.22%	13.81%	
30 to 39	13.90%	11.46%	13.90%	
40 to 49	17.02%	31.44%	17.02%	
50 to 59	17.81%	17.32%	17.81%	
60 to 69	15.06%	12.01%	15.06%	
70 or older	22.40%	12.56%	22.40%	
Sex (%)				0.528
Female	61.35%	66.07%	61.35%	
Race (%)				0.800
White	83.87%	86.95%	83.87%	
Black	11.81%	9.21%	11.81%	
Other	4.32%	3.84%	4.32%	
Metropolitan status: (MSA) (%)	85.79%	92.04%	85.79%	0.140
Geographic region (%)				0.499
Northeast	20.56%	15.99%	20.56%	
Midwest	22.43%	19.88%	22.43%	
South	36.66%	32.54%	36.67%	
West	20.34%	31.59%	20.34%	
Health insurance (%)				0.077
Private	49.94%	59.62%	49.94%	
Medicare	27.04%	24.65%	27.04%	
Medicaid	8.07%	2.99%	8.08%	
Other	14.94%	12.74%	14.94%	

*1. NAMCS only; 2. OPD only; 3. 03-09 NAMCS and 05-09 OPD/ED*.

a *p-values based on adjusted Wald tests with H_0_, Var|_if ME/CFS_ = Var|_if NON−ME/CFS_; Column percentages were listed in the table*.

### ME/CFS Patient Visit Trend, 2000–2009

**Figure 1:**
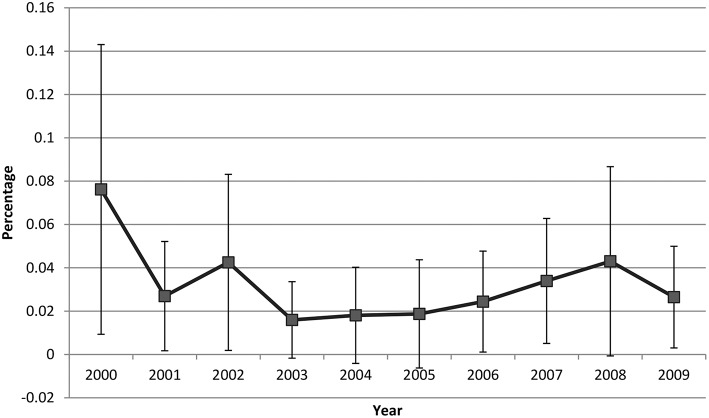
ME/CFS patient visit trend, 2000−2009.

Overall 0.03% of ambulatory visits by patients aged 18 years or older were made by ME/CFS patients. The percentage of ME/CFS-related visits was 0.04% for physician office visits, 0.02% for OPD visits, and 0.01% for ED visits. These proportions did not change significantly over time. The percentage of ME/CFS-related visits ranged from 0.02 to 0.08% during 2000–2009, and there was no statistical evidence for a linearly increasing trend across year (*p*-value for the linear trend: 0.31) (Figure 1).

### Vital Signs, Continuity of Care, Providers Seen, and Visit Disposition

Table 2 summarizes the information on vital signs, continuity of care, major reason for visits, providers seen, and visit disposition of ME/CFS and non-ME/CFS patients. The vital signs of patients visiting for ME/CFS and all others did not differ significantly. The number of visits during the past 12 months made by established ME/CFS patients was greater than that by non-CFS patients (5.60 vs. 4.35, *p* = 0.202). Of ME/CFS-related visits, the top three reasons for visits were chronic/routine problems (60.85%), new problems (14.86%), and chronic/flare up problems (11.47%). Physicians provided care for over 90% of the patient visits for both ME/CFS-related and non-ME/CFS visits. The rates of referral to other physicians did not differ significantly between ME/CFS-related and non-ME/CFS visits.

**Table 2:** Vital sign and continuity of care ambulatory adult visits in USA, 2000–2009.

**Variables**	**All visits**	**ME/CFS visits**	**Non-ME/CFS visits**	***P*-value^**a**^**
Unweighted no. of visits	748,464	130	748,334	
Weighted no. of visits	9,061,664,246	2,911,161	9,058,753,085	
Vital sign				
Body Mass Index (BMI)^1^, mean (SD)	29.13 (7.18)	27.42 (4.60)	29.13 (7.18)	0.274
Body Mass Index (BMI)^1^, median (range)	28.19 (8.14–98.91)	28.25 (19.66–51.58)	28.19 (8.14–98.91)	
Blood Pressure (systolic)^2^, mean (SD)	128.22 (19.52)	126.38 (11.96)	128.22 (19.52)	0.607
Blood Pressure (systolic)^2^, median (range)	129 (0–290)	121.5 (89–180)	129 (0–290)	
Blood Pressure (diastolic)^2^, mean (SD)	76.16 (11.97)	75.72 (6.97)	76.16 (11.97)	0.803
Blood Pressure (diastolic)^2^, median (range)	76 (0–190)	76 (37–109)	76 (0–190)	
Current tobacco user^3^ (%)	11.51%	12.77%	11.51%	0.811
Continuity of care				
Established patient^4^ (%)	87.04%	87.17%	87.04%	0.981
# of visits^5^, mean (SD)	4.35 (6.29)	5.60 (3.80)	4.35 (6.29)	0.202
# of visits^5^, median (range)	2 (0–99)	4 (0–21)	2(0–99)	
Prior-visit status^6^				0.144
None	8.64%	3.60%	8.64%	
1–2 visits	34.36%	29.53%	34.37%	
3–5 visits	24.43%	17.04%	24.43%	
6 or more visits	32.57%	49.83%	32.57%	
New patient^4^				0.306
Referred for this visit (%)	17.01%	10.08%	17.02%	
Not referred for this visit (%)	33.34%	44.86%	33.34%	
Unknown if referred (%)	49.65%	45.06%	49.65%	
Major reason for this visit^4^				0.007
Chronic problem, routine	35.21%	60.85%	35.21%	
New problem	32.17%	14.86%	32.18%	
Chronic problem, flare up	9.08%	11.47%	9.08%	
Preventive care	16.15%	9.55%	16.15%	
Pre-/Post-surgery	7.39%	3.26%	7.39%	
Providers at this visit				
Physician	94.21%	91.14%	94.21%	0.570
Physician assistant	3.94%	2.62%	3.94%	0.434
Nurse practitioner/Midwife	2.10%	0.00%	2.10%	0.000
RN/LPN	34.09%	29.79%	34.09%	0.537
Other	23.57%	32.09%	23.57%	0.284
Mental health provider^7^	0.55%	0.00%	0.55%	0.002
Visit disposition^3^				
Refer to other physician	8.06%	9.89%	8.06%	0.698

*1. 05-09 NAMCS/OPD; 2. 03-09 NAMCS/OPD/ED; 3. 01-09 NAMCS/OPD; 4. 00-09 NAMCS/OPD; 5. 01-09 NAMCE/OPD and 07-09 ED; 6. 07-09 NAMCS/OPD/ED; 7. 07-09 NAMCS/OPD*.

a *p-values based on adjusted Wald tests with H_0_, Var|_if ME/CFS_ = Var|_if NON−ME/CFS_; Column percentages were listed in the table*.

### ME/CFS and Other Chronic Conditions as Co-morbidities

Table 3 shows chronic conditions recorded at ME/CFS-related and non-ME/CFS visits. The co-morbidity rate was higher in visits by patients with ME/CFS than those by patients without ME/CFS (71.30 vs. 61.18%). The most frequent chronic conditions among ME/CFS-related visits were depression (35.79%), hypertension (31.14%), diabetes (20.30%), arthritis (14.11%), and asthma (13.79%).

**Table 3:** ME/CFS and co-morbid conditions.

**Variables**	**ME/CFS visits**	**Non-ME/CFS visits**	***P*-value^**a**^**
Unweighted no. of visits	130	748,334	
Weighted no. of visits^1^	1,354,662	4,307,118,192	
Chronic Conditions			
# of chronic conditions, mean (SD)	1.49 (1.05)	1.27 (1.40)	0.406
# of chronic conditions, median (range)	1 (0–6)	1 (0–13)	
# of chronic conditions (%)			0.343
No chronic condition	28.70%	37.82%	
1 chronic condition	19.19%	27.47%	
2 or more chronic conditions	52.11%	34.71%	
Depression	35.79%	10.67%	0.019
Hypertension	31.14%	30.66%	0.965
Diabetes	20.30%	13.27%	0.500
Arthritis	14.11%	15.83%	0.799
Asthma	13.79%	5.54%	0.239
Chronic obstructive pulmonary disease	12.90%	4.52%	0.372
Hyperlipidemia	11.58%	17.65%	0.443
Cancer	6.59%	6.92%	0.945
Cerebrovascular disease	1.64%	2.05%	0.808
Obesity	1.43%	8.13%	0.002
Osteoporosis	0.11%	3.18%	<0.001
Congestive heart failure	0.06%	2.20%	<0.001
Chronic renal failure	0.00%	1.84%	<0.001
Ischemic heart disease	0.00%	5.10%	<0.001

*1. 05-09 NAMCS/OPD*.

a *p-values based on adjusted Wald tests with H_0_, Var|_if ME/CFS_ = Var|_if NON−ME/CFS_; Column percentages were listed in the table*.

### Healthcare Services and Quality

Table 4 compares quality of healthcare between ME/CFS-related visits and non-ME/CFS related visits. Physicians spent more time for ME/CFS-related visits than non-ME/CFS visits (23.62 vs. 19.38 min, *p* = 0.065). Fewer health counseling services were provided in ME/CFS-related visits than non-ME/CFS related visits (0.16 vs. 0.32 services per visit, *p* = 0.126). The most common counseling services provided during visits by ME/CFS patients were diet/nutrition (8.33%), exercise (8.21%), and smoking cessation (7.24%). Compared to non-ME/CFS visits, a lower proportion of ME/CFS-related visits was provided health education services on stress management (0.75 vs. 3.14%, *p* = 0.010), weight reduction (0.88 vs. 4.02%, *p* = 0.002), injury prevention (0.04 vs. 1.64%, *p* < 0.001), and family planning/contraception (0.17 vs. 1.45%, *p* = 0.037). Smoking cessation (tobacco use/expose) counseling was more prevalent in ME/CFS-related visits than non-ME/CFS visits (7.24 vs. 2.89%, *p* = 0.386). Contrary to health counseling services, more diagnostic/screening tests were provided in ME/CFS-related visits than non-ME/CFS visits (1.00 vs. 0.50 services per visit, *p* = 0.132). The most common diagnostic/screening tests provided during visits by ME/CFS patients were CBC (25.01%), glucose (19.05%), and lipids/cholesterol (12.84%), but did not reach any statistical significance level of 0.05. Non-medication treatment was more frequently provided at ME/CFS-related visits than non-ME/CFS visits (0.15 vs. 0.07 per visit, *p* = 0.244).

**Table 4:** Quality indicators of healthcare between ME/CFS-related and non-ME/CFS related visits.

**Variables**	**ME/CFS visits**	**Non-ME/CFS visits**	***P*-value^**a**^**
Unweighted no. of visits	130	748,334	
Weighted no. of visits	1,354,662	4,307,118,192	
Time Spent with Physician^1^, mean (SD)	23.62 (14.86)	19.38 (13.83)	0.065
Time Spent with Physician^1^, median (range)	20 (0–90)	15 (0–240)	
Health Education Services^2^			
# of services ordered, mean (SD)	0.16 (0.57)	0.32 (0.74)	0.126
# of services ordered, median (range)	0 (0–3)	0 (0–5)	
None (%)	92.00%	80.30%	
One (%)	0.21%	11.05%	
2 or more (%)	7.79%	8.65%	
Type of Health Education Services			
Diet/Nutrition^3^	8.33%	12.48%	0.306
Exercise^3^	8.21%	9.79%	0.677
Weight reduction^4^	0.88%	4.02%	0.002
Stress management^5^	0.75%	3.14%	0.010
Tobacco use/exposure^5^	7.24%	2.89%	0.386
Growth/Development^3^	2.97%	0.54%	0.412
Asthma education^4^	2.38%	0.91%	0.531
Injury prevention^7^	0.04%	1.64%	<0.001
Other health education^5^	23.74%	19.86%	0.702
Family planning/Contraception^8^	0.17%	1.45%	0.037
Diagnostic/Screening Services^9^			
# of services ordered, mean (SD)	1.00 (1.51)	0.50 (1.06)	0.132
# of services ordered, median (range)	0 (0–5)	0 (0–5)	
None (%)	57.73%	76.74%	
One (%)	20.32%	8.77%	
2 or more (%)	21.95%	14.49%	
Blood tests ordered: (%)			
CBC^10^	25.01%	15.03%	0.191
Glucose^11^	19.05%	8.63%	0.248
HgbA1c^12^	3.24%	3.53%	0.916
Lipids/Cholesterol^13^	12.84%	8.11%	0.330
Other blood test^14^	33.94%	15.12%	0.065
Non-Medication Treatment^5^			
# of treatments ordered, mean (SD)	0.15 (0.36)	0.07 (0.27)	0.244
# of treatments ordered, median (range)	0 (0–2)	0 (0–3)	
None (%)	84.78%	93.72%	
One (%)	15.17%	5.80%	
2 or more (%)	0.06%	0.48%	
CAM^7^	8.55%	1.09%	0.125
Physical therapy^5^	2.70%	2.65%	0.984
Psychotherapy^3^	0.61%	2.24%	0.003
Other mental health counseling^5^	0.06%	1.47%	<0.001

*1. 00-09 NAMCS and 00 OPD; 2. 05-09 NAMCS/OPD; 3. 00-09 NAMCS/OPD; 4. 01-09 NAMCS/OPD; 5. 05-09 NAMCS/OPD; 6. 05-09 NAMCS/OPD; 7. 00, 05-09 NAMCS/OPD; 8. 00 & 09 NAMCS/OPD; 9. 05-09 NAMCS/OPD and 03-04 ED; 10. 01-09 NAMCS/OPD and 00-09 ED; 11. 03-09 NAMCS/OPD and 01-09 ED; 12. 03-09 NAMCS/OPD and 01-04 ED; 13. 00-09 NAMCS/OPD and 01-04 ED; 14. 00, 05-09 NAMCS/OPD and 00, 03-09 ED*.

a *p-values based on adjusted Wald tests with H_0_, Var|_ifCFS_ = Var|_ifNON−CFS_; Column percentages were listed in the table*.

## Discussion

To our best knowledge, this is the first study that used a nationally representative healthcare sample of the U.S. to investigate the visit trend of diagnosing ME/CFS over years. This paper examined demographics, co-morbidities, and healthcare for visits by ME/CFS patients using a nationally representative sample of patient visits to physician offices, hospital outpatient departments, and emergency departments from 2000 to 2009. We found the overall estimated percentage of ME/CFS-related visits to be 0.03% with no statistical evidence (p-trend = 0.31) for a decline or increase from 2000 to 2009. Assuming no repeat visits by same patients, the percentage estimate of visits by ME/CFS patients would approximately reflect the prevalence estimates reported from previous studies in primary care settings.

Visits by ME/CFS patients report slightly more co-morbid conditions than visits by patients without ME/CFS. Over 70% of visits by ME/CFS patients report one or more co-morbid conditions, adding to complexity in ME/CFS healthcare. Our results on healthcare for visits by ME/CFS patients are mixed. While physicians spent more time during visits by ME/CFS patients than that by patients without ME/CFS, a lower portion of visits by ME/CFS patients was provided counseling for diet/nutrition, exercise, and weight reduction. On the other hand, a higher portion of visits by ME/CFS patients were provided diagnostic/screening tests and non-medication treatment twice as often as visits by non-ME/CFS patients.

### Conclusion

In conclusion, we found that there is no increasing or decreasing trend in the percentage of ME/CFS-related visits during 2000–2009. Compared to visits by non-ME/CFS patients, visits by ME/CFS patients are provided more direct care time by physicians, more diagnostic/screening tests, and more non-medication treatments but less health counseling services. Future research should consider developing basic guidelines or recommendations for appropriate healthcare in ME/CFS-diagnosed visits such as providing weight reduction or nutrient/diet counseling for ME/CFS patients with greater BMI. When providing exercise counseling, one should be cautious of the impact of exercise. An individualized exercise or activity management plan should be emphasized to balance rest and activity to avoid post-exertional malaise flare-ups. Before starting any individualized exercise or activity management program, one should be carefully assessed and monitored periodically on their muscle strength and functional status for any physical activity.

It will also allow for identifying the potential interrupted time series that might be resulted from future ICD coding transition/change and the impact of the 2015 IOM recommendation on ME/CFS. Future investigation on this topic is warranted.

### Study Limitation

Several limitations of this study should be mentioned. First, NAMCS, and NHAMCS do not include identifiers for individual patients; therefore, some patients who made visits more than once may have had their visits counted independently, which would yield inaccurate estimates of the variance. The surveys used a randomly selected sampling unit (a physician or facility) with a reporting period of the randomly assigned 1-week for NAMCS and 4-week for NHAMCS. Although our results showed that on average ME/CFS patients made about 5.6 visits per year and that translated into one visit every 2 months, it's less likely that the same patient would visit to the same selected physician or facility during the randomly assigned 1-week reporting period. Therefore, we believe that this limitation might affect our conclusion only to a small degree. Second, the NAMCS and NHAMCS included at most only three diagnosis codes and chief complaints. A greater number of listed diagnosis codes was associated with a higher likelihood of identifying ME/CFS. Thus, we may not have been able to identify some ME/CFS patients who were diagnosed as ME/CFS in 4th or later diagnosis. There is an ICD-9-CM code 780.79 for “Other malaise and fatigue” which converts approximately to ICD-10-CM G93.3 for Postviral fatigue syndrome ([Benign] myalgic encephalomyelitis) but 780.79 was not commonly documented in this data source. Therefore, in our analysis we focused on visits with ICD-9-CM code, 780.71 for chronic fatigue syndrome. One should also be aware that the ME/CFS related visits might be under-reported due to the possibility of a substantial level of omissions by healthcare providers. Finally, due to the small number of cases identified one should be cautious of generalizing the results beyond the general healthcare setting.

## Ethics Statement

This is a secondary analysis using publicly available data. This analysis was based on the National Ambulatory Medical Care Survey (NAMCS) (33) and the National Hospital Ambulatory Medical Care Survey (NHAMCS) (34) from 2000 to 2009. Since 1992, both surveys have been administrated by the National Center for Health Statistics (NCHS), Centers for Disease Control and Prevention (CDC) (35–38). The NCHS Research Ethics Review Board approved the protocols for both the NAMCS and NHAMCS surveys, including a waiver of the requirement for informed consent of participating patients.

## Author Contributions

JML had the original idea for the study. JML developed the analysis plan and JB performed all statistical analyses with guidance and support from JML. JB and JML drafted the paper and contributed to the interpretation and the final manuscript.

### Conflict of Interest Statement

The authors declare that the research was conducted in the absence of any commercial or financial relationships that could be construed as a potential conflict of interest.
